# Productivity change and its driving forces in Chinese healthcare sector

**DOI:** 10.1371/journal.pone.0243460

**Published:** 2020-12-11

**Authors:** Zhensheng Chen, Xueli Chen, Tomas Baležentis, Xiaoqing Gan, Vivian Valdmanis

**Affiliations:** 1 School of Management, Nanchang University, Nanchang, China; 2 Anhui University of Finance and Economics, Bengbu, China; 3 Chinese Academy of Social Sciences, Beijing, China; 4 Lithuanian Institute of Agrarian Economics, Vilnius, Lithuania; 5 Western Michigan University, Kalamazoo, MI, United States of America; Institute for Advanced Sustainability Studies, GERMANY

## Abstract

Since the last medical reform in 2009, China’s public hospitals have been facing the changes in the institutional environment. However, the effects of reforms have not been received enough attention to deliver evidence-based implications. In this paper, we first assess the efficiency of regional public hospitals from 2011 to 2018, employing a proposed method based on an additive indicator and an aggregate directional distance function (DDF). The method applied allows for decomposing total factor productivity (TFP) indicator into three components, including technical efficiency change (TEC), total productivity (TP) and scale efficiency change (SEC). Second, following the efficiency assessment, we carry post-efficiency analysis to identify the determinants of efficiency of the public hospitals. The results show that annual average TFP growth rate is 1.38%, which is driven mainly by TEC. Regional disparities of public hospitals’ performance are expanding. Almost 75% of the regions considered show a positive TFP growth. The regression results show that the significant determinants of efficiency of regional public hospitals include the price of and demand for health services.

## Introduction

The key goal of a healthcare system is to provide equitable and accessible healthcare services efficiently. As the major provider of healthcare services in China, public hospitals play an important role in the medical system, not only accounting for 54.69 percent of hospitals and 85.84 percent of visits, but also incurring the majority of healthcare expenditure [1]. With the increasing population size, the aging of population, the growing per capita income, the demand for medical services is rising rapidly. In the period of rapid economic growth in China, the government's investment in the health sector have been used to establish new public hospitals and expand the scale of public hospitals to meet the growing demand for medical services. Since China's economy enters the "new normal", the absolute investment of the Chinese government in the health sector is still growing, but the growth rate is gradually decreasing [1], resulting in the impossibility to increase the supply of medical services by means of large-scale investments as in the past. Thus, increasing supply by improving efficiency is a more realistic approach.

Healthcare service delivery system operated inefficiently before China implemented the third round of reform on health system in 2009 [2]. The inefficiency was reflected by the rapidly increasing health expenditure and low access to the healthcare services in China. Different from the earlier medical reform, the last reform emphasizes the government's responsibility to public hospitals, instead of putting public hospitals into the market as for-profit hospitals. After 2009, the government carried out several reforms on public hospitals, covering the compensation mechanism, the organizational structure, the functional positioning, and the operation mechanism. Assessment of the productivity and efficiency of China’s public hospital system may provide evidence on the success of the 2009 reform.

The preferred tools to analyze efficiency of healthcare providers include Data Envelopment Analysis (DEA) that can be supplemented by Malmquist indices and distance functions [3]. As a non-parametric method, DEA has many advantages that the parametric method does not have. For example, DEA only assumes some basic axioms of production theory that are satisfied easily, without assuming particular functional form of the production function [4]. One of the well-known drawbacks of DEA is that slacks are not included in the efficiency scores, which means that a DMU may still have a performance gap in the presence of full efficiency score (measured in the radial manner). The additive directional distance function (DDF) can address the aforementioned shortcomings.

The aim of this paper is to identify the patterns of TFP growth and its correlates in the wake of China's recent healthcare reform with focus on the public hospitals. The paper contributes to the existing literature in that it applies the additive indicator and an aggregate directional distance function. This allows gaining additional insights into the aggregate performance of the public hospitals. The additive indicator identifies the contribution of each DMU to the total productivity change and overcomes some drawbacks of DEA related to weak efficiency. Second, we take regional units as observations. This provides a more comprehensive approach towards effects of public hospital reform in different regions. The sample used covers 31 regions in China from 2011 to 2018.

### Literature review

Efficient use of medical resources is an important problem faced by each country's medical system and benchmarking is often the only way to identify the performance gaps. China implemented three rounds of reforms on the medical system in order to boost its performance. In Table 1, we briefly describe these reforms. During the planned economy stage the public hospitals were financed by budget and acted on executive orders. The market stage meant that public hospitals were authorized to operate independently out of government's control and were mainly self-financed. The latest healthcare reform of 2009 intended to promote the social benefit and efficiency of the public hospitals through reshaping the compensation mechanism, organizational structure and payment mode.

**Table 1:** The main actions, achievements and problems in the three stages of Chinese public hospitals reform (1949–2020).

Planned economy stage (1949–1978): government as health provider directly financed public hospitals.	
**Main Actions**	After the founding of new China, medical resources were scarce. In order to quickly establish a medical system covering the whole people, public hospitals were financed mainly by government, and provided free or cheap health service for mass.
**Achievements**	Establishment of a national medical system; Improvement of medical conditions; Increase of life expectancy.
**Problems**	High budget pressure; Low production efficiency and quality of medical services.
Marketization stage (1978–2009): government relaxed the regulation on public hospitals and reduced subsidies to public hospitals. Public hospitals were mainly self-financed.	
**Main Actions**	Market mechanism was introduced into public hospitals to improve medical conditions, improve the quality of medical services and production efficiency of public hospitals, and reduce the pressure on the budget.
**Achievements**	Medical resources enriched; Advanced medical equipment and technology widely used.
**Problems**	The heavy burden of patients due to rapidly rising medical expenses; More attention to treatment than prevention resulted in the low utilization efficiency of medical resources; Public hospitals operated as for-profit organizations
The latest medical reform (2009–2020): The government has the leading responsibility in the medical system. Public hospitals are public welfare and medical services are welfare.	
**Main Actions**	The social benefits and the health management role of public hospitals are emphasized; The efficiency of public hospitals can be improved and the rapidly increase in health expenditure is controlled through the reforms on compensation mechanism, organizational structure and payment mode.
**Achievements**	The proportion of financial subsidies in medical expenditure increased; improvement in the quality of medical and health services.
**Problems**	The effectiveness of the reform is unknown.

As this paper focuses on the outcomes of the most recent medical reform of 2009, a detailed description thereof is given in Table 2. One can note that five main measures were implemented during this reform: 1) construction of medical security system with managerial mechanism for all residents; 2) establishment of medicine system that improves medicine management; 3) improvement of primary medical and health services by allocates more resources to the primary medical units; 4) promotion of urban-rural equality in terms of access to health services; 5) improvement of operations and management of public hospitals in terms of public welfare.

**Table 2:** The measures taken during China's recent medical reform of 2009.

Element	Measures
Basic medical security system	Expanding the coverage of the basic medical security, improving the level of basic medical security, and reducing the burden of medical expenses paid by the patients. Improving the negotiation mechanisms between medical insurance agencies and medical service providers, and reforming payment methods. Determining the payment standards for drugs, medical services and medical materials, controlling the medical costs.
National essential drug system	Setting up a list of essential drugs and revising it regularly. Establishing a system for prioritization and rational use of essential drugs.
Primary medical and health service system	Improve the rural three-level medical and health service network, transform the operating mechanism of primary medical and health institutions. Establish an assessment and incentive system that considers service quality and quantity and responsibility.
Equalization of basic public health services	Basic public health services cover urban and rural areas. Improve public health service capacity.
Pilot reform of public hospitals	Reform the management system, operation mechanism, and supervision mechanism of public hospitals, so that public hospitals adhere to the principles of maintaining public welfare and social benefits, and focus on patients. Reform the compensation mechanism of public hospitals, and change the compensation of public hospitals from three channels of service charges, drug markup income and financial subsidies to two channels of service charges and financial subsidies. Provide special subsidies for public health tasks undertaken by public hospitals, facilitate preferential investment to the government-designated public service (emergency treatment, foreign aid, agricultural support, and border support). Transform some public hospitals into private medical institutions; encourage private capital to establish non-profit hospitals.

China relies on the basic medical insurance, which became the largest healthcare service buyer. It allows patients to access medical treatment without a delay due to high medical expenditures. In addition, China adjusted the revenue structure of public hospitals through cancelation of drug markup, increase in subsidy and improvement to the pricing mechanism of medical services. Similar reforms are being carried out in other countries or regions to overcome the inefficiency.

The efficiency of medical institutions has been analyzed in many regions, such as Italy [5], Norway [6], America [7], Canada [8, 9], India [10], Austria [11], China [12–14]. There have been studies on the efficiency in health industry that were carried out at the micro level (i.e., individual medical institution were used as the DMUs). Jat and San [10] evaluated the technical efficiency of 40 public district hospitals based on DEA and found that 50% of sample were technically efficient. Ajlouni, et al. [15] used DEA and Pabon-Lasso Diagram to assess the performance efficiency of 15 public hospitals in Jordan. Sultan and Crispim [16] evaluated efficiency of 27 general public hospitals in Jordan using DEA from 2010 to 2014 and sorted them with regards to the underlying performance patterns. Rego et al. [17] employed DEA to investigate the efficiency of 59 public Portuguese hospitals and found that the introduction of market principles and changes in organizational structure had a positive impact on Portuguese public hospitals. Bin et al. [12] measured efficiency of 19 tertiary general public hospitals in Tianjin based on DEA and found that more than half of the sample operated at full technical and scale efficiency. Xu et al. [18] employed the ratio analysis, stochastic frontier analysis, and DEA to examine the efficiency of 50 tertiary public hospitals and found that the efficiency of specialized hospitals was higher than that of general hospitals and traditional Chinese medicine hospitals.

Another strand of the literature on efficiency in health industry takes regions as DMUs. Sulku [19] employed DEA and Malmquist index to measure the productivity change of public hospitals before and after the Health Transformation Programme reform in Turkey and found that the productivity was improved. Afonso and St. Aubyn [20] used DEA to assess health efficiency of 30 OECD countries and regressed efficiency scores on environmental variables. Hu et al. [14] used DEA to investigate the regional hospital efficiency in China before and after health insurance reform of New Rural Cooperative Medical System and showed that hospital efficiency increased. Chen et al. [21] decomposed the productivity growth to analyze regional medical institutions operations based on an aggregate DDF and found that TFP growth (at the annual rate of 1.87%) in Chinese medical institutions was mainly driven by technological progress. Shen and Valdmanis [22] adopted an aggregate DDF to identify the contribution to hospital performance among Chinese regions and found that technical inefficiency dominated the overall inefficiency.

In addition, the efficiency changes were explained from different aspects. Hu et al. [14] investigated the impact of health insurance reform on the efficiency of hospitals in China. Sheikhzadeh et al. [23] found that public hospitals were generally relatively more efficient than private ones and suggested to divest some inefficiently used assets. Cavalieri et al. [5] employed the two-stage DEA to investigate the impact of Per Case Payment System on the technical efficiency of the Italian hospital sector. Biørn et al. [6] examined the impact of the financial reform-ABF on hospital efficiency in Norway. Cho et al. [7] employed the two-stage approach to determine the impact of health information technology adoption and hospital-physician integration on hospital efficiency. Czypionka et al. [11] applied DEA to examine the impact of ownership type on efficiency. Chilingerian and Sherman [24] took the impact of quality on efficiency into consideration.

In this paper, we will follow the regional perspective and consider the provinces of China as DMUs when evaluating the efficiency of public hospitals. Following Chen et al. [21] and Shen and Valdmanis [22], we employ the aggregate DDF and construct the additive Luenberger-Hicks-Moorsteen (LHM) TFP indicator, which satisfies the conditions for completeness defined by O'Donnell [25] and Kerstens et al. [26]. This indicator can be decomposed into technical efficiency change, scale efficiency change, and technological change. Finally, we regress efficiency scores on influencing factors to explain the productivity change changes.

## Methods

### Production technology and distance functions

The production possibility set (*T*) defined the production technology. Suppose *N* inputs (denoted by *x*) are used to produce *M* outputs (denoted by *y*) during the operation of the public hospitals. The production technology *T* is formally defined as

$$T=\left\{(x,y)\in {\mathbb{R}}_{+}^{N+M}:\;x\;can\;produce\;y\right\}.$$ (1)

Hackman [27] presented the following economic axioms that are imposed on the DEA production technology: *T* is a closed set, *T* is convex, and free disposability of inputs and outputs is maintained.

Distance functions are used to measure the distances between the evaluated unit and the benchmark point on the production frontier. The directional distance function proposed by Chambers et al. [28] measures the performance gap for a given direction:

$$D(x,y;g_{x}^{},g_{y}^{})=\max\left\{\delta ,\theta \in {\mathbb{R}}_{+}:(x-\delta g_{x}^{},y+\theta g_{y}^{})\in T\right\},$$ (2)

where *δ* is the inefficiency score related to the input use, measuring the potential decrease in inputs and *θ* is the output inefficiency indicating the possible improvement in outputs. In order to identify the contribution of each region to the overall inefficiency of China's health care system the aggregate directional distance function can be applied. The aggregate DDF measures inefficiency in terms of the aggregate input and output quantities comprising of all the DMUs [29, 30]. The common direction is used which is defined as sum of inputs and outputs:

$$(g_{x}^{},g_{y}^{})=(\sum_{k=1}^{K}g_{x}^{},\sum_{k=1}^{K}g_{y}^{}).$$ (3)

The advantage of the aggregate directional distance function is that it allows aggregating the region-specific inefficiency values (potential decease in inputs and potential increase in outputs) in a reasonable manner.

### The additive LHM-TFP indicator and its decomposition

In this paper, we apply the directional distance function based on additive form [29] to compute the LHM indicators [31]. The LHM indicator for productivity growth between period *t* and *t*+1 is defined with respect to base periods *t* and *t*+1 as follows:

$$\begin{array}{l} LHM^{t}=[D_{VRS}^{t}(x_{k}^{t},y_{k}^{t};0,g_{y}^{t})-D_{VRS}^{t}(x_{k}^{t},y_{k}^{t+1};0,g_{y}^{t+1})]\\ \;-[D_{VRS}^{t}(x_{k}^{t+1},y_{k}^{t};g_{x}^{t+1},0)-D_{VRS}^{t}(x_{k}^{t},y_{k}^{t};g_{x}^{t},0)],\\ LHM^{t+1}=[D_{VRS}^{t+1}(x_{k}^{t+1},y_{k}^{t};0,g_{y}^{t})-D_{VRS}^{t+1}(x_{k}^{t+1},y_{k}^{t+1};0,g_{y}^{t+1})]\\ \;-[D_{VRS}^{t+1}(x_{k}^{t+1},y_{k}^{t+1};g_{x}^{t+1},0)-D_{VRS}^{t+1}(x_{k}^{t},y_{k}^{t+1};g_{x}^{t},0)]. \end{array}$$ (4)

LHM TFP indicator is obtained as the arithmetic mean of the two indicators in Eq 4:

$$TFP^{t,t+1}=\frac{1}{2}\left(LHM^{t}+LHM^{t+1}\right).$$ (5)

Following Ang and Kerstens [32] and Shen et al. [4], LHM-TFP indicator can be decomposed into technical efficiency change (TEC), scale efficiency change (SEC), and technological progress (TP):

$$\begin{array}{l} TFP=TEC+TP+SEC,\\ TEC=D_{}^{t}(x_{k}^{t},y_{k}^{t};0,g_{y}^{t})-D_{}^{t+1}(x_{k}^{t+1},y_{k}^{t+1};0,g_{y}^{t+1}),\\ TP=\frac{1}{2}\left(\begin{array}{l} \left[D_{}^{t+1}(x_{k}^{t},y_{k}^{t};0,g_{y}^{t})-D_{}^{t}(x_{k}^{t},y_{k}^{t};0,g_{y}^{t})\right]\\ +[D_{}^{t+1}(x_{k}^{t+1},y_{k}^{t+1};0,g_{y}^{t+1})-D_{}^{t}(x_{k}^{t+1},y_{k}^{t+1};0,g_{y}^{t+1})] \end{array}\right),\\ SEC=TFP-TEC-TP\\ \;=\frac{1}{2}\left(\begin{array}{l} \left[D_{}^{t}(x_{k}^{t+1},y_{k}^{t+1};0,g_{y}^{t+1})-D_{}^{t}(x_{k}^{t},y_{k}^{t+1};0,g_{y}^{t+1})\right]\\ -[D_{}^{t}(x_{k}^{t+1},y_{k}^{t};g_{x}^{t+1},0)-D_{}^{t}(x_{k}^{t},y_{k}^{t};g_{x}^{t},0)]\\ +[D_{}^{t+1}(x_{k}^{t+1},y_{k}^{t};0,g_{y}^{t})-D_{}^{t+1}(x_{k}^{t},y_{k}^{t};0,g_{y}^{t})]\\ -[D_{}^{t+1}(x_{k}^{t+1},y_{k}^{t+1};g_{x}^{t+1},0)-D_{}^{t+1}(x_{k}^{t},y_{k}^{t+1};g_{x}^{t},0)] \end{array}\right). \end{array}$$ (6)

TEC measures the change in the distance between an observation and the production frontier. A positive TEC indicates that the utilization of resources improves and promotes TFP growth, whereas a negative one indicates that the resources in healthcare service system are being used less efficiently compared to the benchmark. TP refers to the movement of the production frontier of the healthcare service system, which is affected by the technological innovation (e.g., improved medical equipment) or managerial innovations (e.g., organizational structure improvement). SEC indicates changes in the distance from the operation scale of an observation to the optimal production scale. Thus, positive values of SEC indicate that the productivity growth is caused by moving towards region of higher marginal productivity as approaching the optimal production scale. Conversely, the negative values imply that health care system is departing from the optimal production scale and resources are being used with lower productivity.

The application of the aggregate DDF also allows for a meaningful aggregation of the province-specific TFP growth rates [22]. Therefore, the national public hospital system TFP growth is measured as the sum of 31 province-level TFP changes. Formally, this relationship ios given as

$$TFP_{China}^{}=\sum_{k=1}^{K}TFP_{k}^{}.$$ (7)

### Estimation of the distance functions

In this paper, DEA is used to estimate the distance functions [33] via the linear programming. Taking the output-oriented distance function as an example, assuming that the input levels are fixed, the objective function maximizes outputs by a factor of *θ* multiplied by the directional vector. To calculate LHM TFP indicator and its components presented in Eq (6), we need 10 distance functions over different period pairs under variable returns to scale (VRS) assumption. For example, to assess the efficiency of public hospitals system in period t, the output-oriented directional distance function *D*(*x*,*y*;0,*g*_*y*_) is obtained by solving the following problem:

$$\begin{array}{l} \;D(x,y;0,g_{y}^{})=\underset{\theta ,\lambda_{k}}{\max}\theta \\ s.t.\sum_{k=1}^{K}\lambda_{k}y_{k}^{m}\geq y_{}^{m}+\theta g_{y}^{m},\forall m=1,\ldots ,M;\\ \;\sum_{k=1}^{K}\lambda_{k}x_{k}^{n}\leq x_{}^{n},\forall n=1,\ldots ,N;\\ \;\sum_{k=1}^{K}\lambda_{k}=1;\\ \;\lambda_{k}\geq 0,\forall k=1,\ldots ,K \end{array}$$ (8)

where *λ* and *θ* are activity variables and inefficiency score respectively. A positive *λ* indicates that corresponding DMU is selected as a reference when defining the benchmark on the production frontier; *θ* represents the potential for increase in output. The convexity constraint $\sum_{k=1}^{K}\lambda_{k}=1$ implies the assumption of VRS.

The input-oriented directional distance function *D*(*x*,*y*;*g*_*x*_,0) is obtained by solving the following problem:

$$\begin{array}{l} \;D(x,y;g_{x}^{},0)=\underset{\delta ,\lambda_{k}}{\max}\delta \\ s.t.\sum_{k=1}^{K}\lambda_{k}y_{k}^{m}\geq y_{}^{m},\forall m=1,\ldots ,M;\\ \;\sum_{k=1}^{K}\lambda_{k}x_{k}^{n}\leq x_{}^{n}-\delta g_{x}^{n},\forall n=1,\ldots ,N;\\ \;\sum_{k=1}^{K}\lambda_{k}=1;\\ \;\lambda_{k}\geq 0,\forall k=1,\ldots ,K \end{array}$$ (9)

where *δ* is inefficiency score, indicating the potential for decrease in inputs. It should be noted that the direction vector of this paper is defined as aggregate output/input values of the whole public hospitals system.

## Data

Regional public hospitals datasets (2011–2018) from the National Bureau of Statistics of China and Statistical Information Center of the National Health Commission are used to assess the productivity growth of regional public hospitals. Chilingerian and Sherman [24] attached great importance to human resources in the service process of hospitals and suggested to distinguish between different types of personnel. In addition, the number of beds is regarded as a proxy of capital investment. The inputs (measured in 10,000) include number of licensed doctors, number of registered nurses, other technical staff and number of beds while the outputs (measured in millions) include number of outpatient visits, number of inpatients visits, the number of inpatient surgeries, and emergency room visits. The health care reform officially announced on March 17, 2009 obviously required a certain lag period to manifest its effects. Accordingly the period 2011–2018 is chosen for the analysis.

In order to take the socio-economic differences into account, we divide China’s 31 province-level regions into three zones following Shen and Valdmanis [22]. The eastern region includes Beijing, Tianjin, Hebei, Liaoning, Shanghai, Jiangsu, Zhejiang, Fujian, Shandong, Guangdong, and Hainan. The inland region includes Shanxi, Jilin, Heilongjiang, Anhui, Jiangxi, Henan, Hubei, and Hunan. Finally, the western region comprises Inner Mongolia, Guangxi, Sichuan, Chongqing, Guizhou, Yunnan, Tibet, Shannxi, Gansu, Qinhai, Ningxia, and Xinjiang.

Factors explaining the performance of hospitals were categorized into two categories, including market conditions and regulation [14]. With regard to market conditions, children and elderly are likely to exhibit higher demand for health services. Thus, the ratio of population aged 0–14 and above 65 years to the total populations (*Demand*) in a region is used to measure the demand for health service. In general, the increase in demand for medical service leads to more skilled doctors and scale effect, resulting in improved in efficiency of public hospitals. The ratio of third-class hospitals to total hospitals (*Qualia*) in a region is employed to measure the quality of inputs in the market. The combination of advanced medical equipment and skilled staff can provide more healthcare services.

The ratio of the number of for-profit hospitals to total hospitals (*Compet*) in a province reflects the intensity of competition. Ownership will be ignored due to focus on public hospitals. Expenditure per outpatient visit (*Oprice*) measures the price of outpatient services while expenditure per inpatient visit (*Iprice*) measures the price of inpatient service. *Subsid* represents government's responsibility in medical service, which is measured by the ratio of government subsidy to the public hospital income. Due to the differences in medical insurance plans, the ratio of the insured of basic medical insurance to population in a region is adopted to reflect the reform of medical insurance plan.

The descriptive statistics for inputs/outputs and the explanatory variables are given in Tables 3 and 4 respectively.

**Table 3:** Descriptive statistics for inputs and outputs (2011–2018).

Indicators		Mean	S.D.	Min	Max
Inputs	Licensed Doctors	9.68	6.29	0.40	29.04
	Registered Nurse	10.15	6.79	0.17	33.46
	Other technical staff	5.41	3.32	0.36	14.43
	Beds	21.95	14.24	0.84	60.85
Outputs	Emergency Treatment	231.80	182.12	9.60	825.89
	Outpatient	10.90	9.21	0.44	42.29
	Inpatients	6.71	4.64	0.15	19.16
	Surgeries	1.48	1.15	0.02	7.35

**Table 4:** Descriptive statistics and definitions of the efficiency determinants.

Variable	Definition	Mean	S.D.	Min	Max
*Oprice*	expenditure per outpatient visit, RMB	218.19	62.42	75.40	530.90
*Iprice*	expenditure per inpatient visit, RMB	8574.64	3241.69	3906.30	22645.80
*Insura*	ratio of insured of basic medical insurance to population	0.47	0.25	0.13	0.98
*Subsid*	ratio of government subsidy to the public hospital income	0.33	0.09	0.20	0.72
*Compet*	ratio of the number of for-profit hospitals to total hospitals	0.48	0.15	0.05	0.78
*Qualia*	ratio of third-class hospitals to total hospitals	0.08	0.03	0.02	0.16
*Demand*	ratio of population aged 0–14 and above 65 years	0.26	0.04	0.16	0.34

## Empirical results

The average annual TFP growth rates and its decomposition for China’s regions from 2011 to 2018 are shown in Table 5. The second column shows that the average annual productivity growth (1.38%) for the entire sample is mainly driven by TEC (0.65%), which implies that resources in public hospitals have been exploited in a more reasonable manner. At the regional level, the inland provinces show the highest TFP growth, whereas the lowest on eis observed for the western zone. The results indicate that the TFP growth in eastern and inland zones is mainly driven by TP, implying that the overall improvement in management of public hospital is pushing the production frontier outwards. The TFP growth in western zone is driven mainly by SEC. thus, the changes in the operation scale in the western zone are the most important in the sense if productivity growth. SEC in the inland zone shows a negative contribution towards productivity growth, which implies that the scale of public hospitals in inland zone has been departing from the optimal one.

**Table 5:** The average annual TFP growth rates and its decomposition in China’s regions (%, 2011–2018).

	China	Eastern	Inland	Western
**TFP**	1.38	0.44	0.59	0.35
**TEC**	0.65	0.16	0.32	0.17
**TP**	0.60	0.26	0.37	-0.03
**SEC**	0.13	0.02	-0.10	0.21

**Fig 1:**
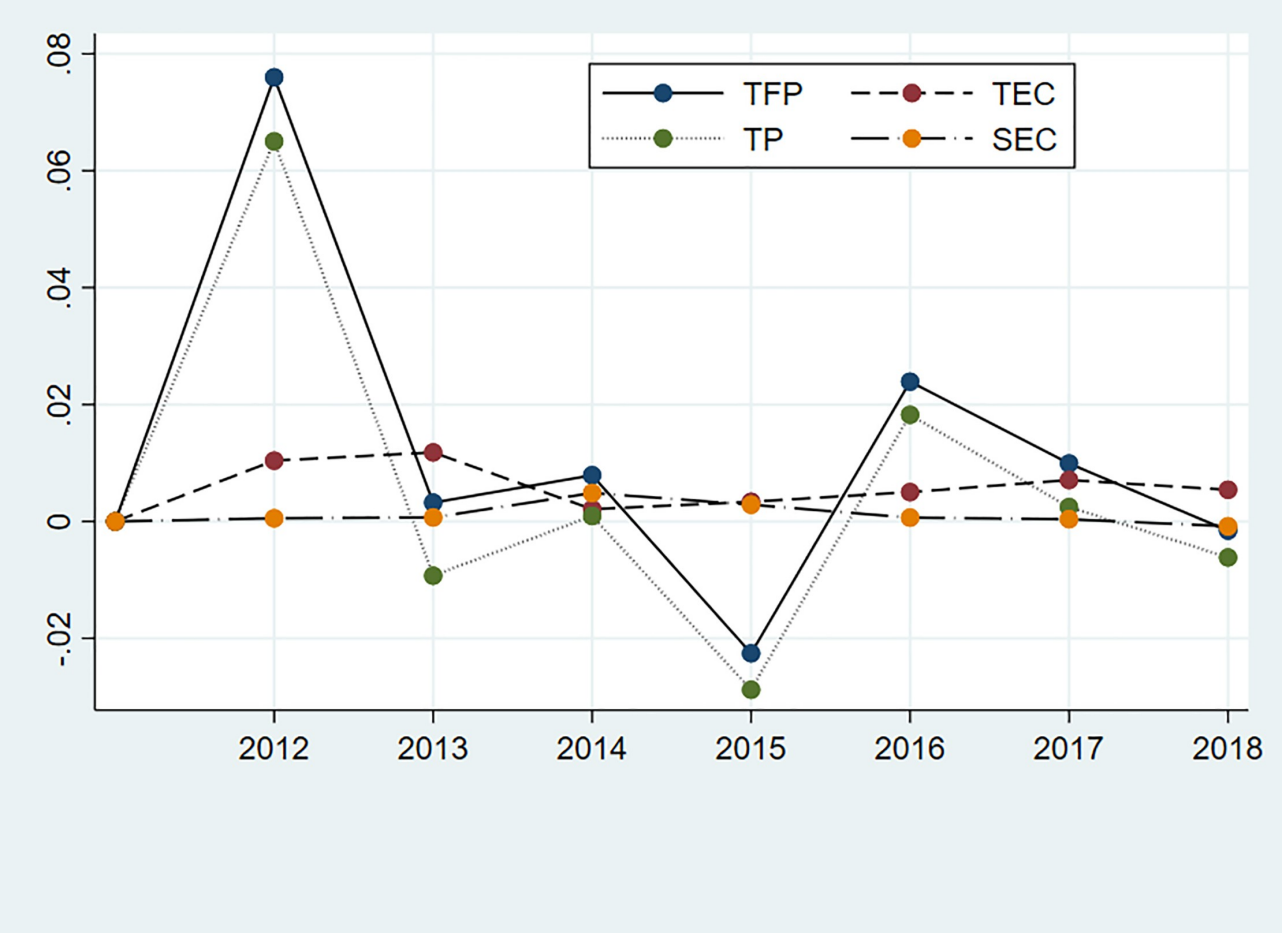
TFP growth of Chinese public hospital system across the provinces, 2012–2018.

We find that the performance disparities of regional public hospitals have been increasing during 2011–2018, as shown in Fig 1. The minimum TFP growth rate of regional public hospitals was slightly positive in 2011, implying the entire public hospitals system was improving. The variation of the TFP growth among the regions increased with time. Therefore, some regions kept improving their performance, whereas others declined in terms of the TFP growth. Still, the lower quartile of the TFP growth rate remained slightly positive from 2013 to 2018, which indicates that 75% of regions considered managed to improve the TFP of their public hospitals. What is more, the proportion remained rather stable.

**Fig 2:**
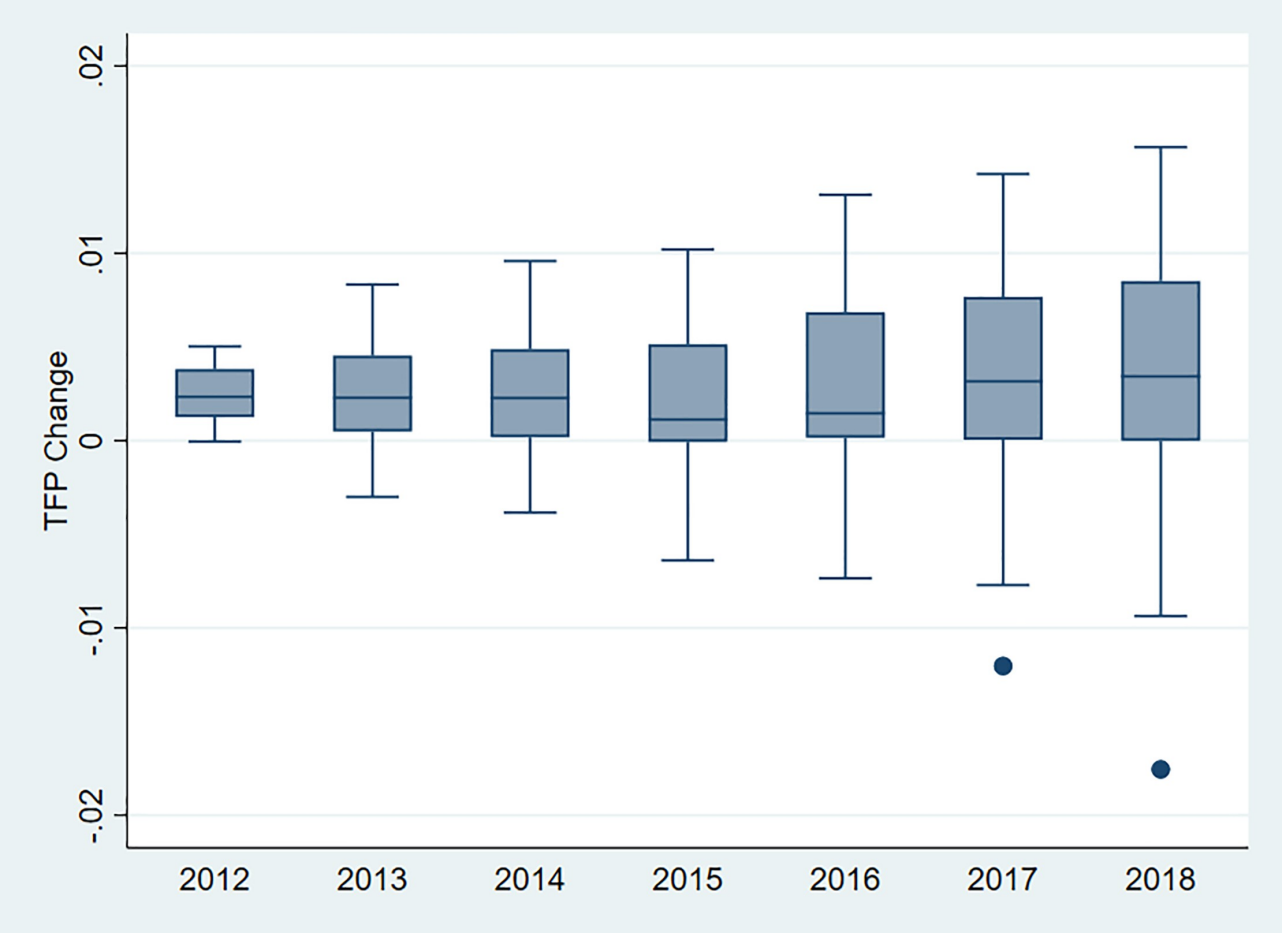
Evolution of TFP indicators and its components (2011–2018).

Evolution of TFP indicators and its components from 2011 to 2018 are presented in Fig 2. The TFP growth is followed by the TFP decrease from 2011 to 2017, which finally results in little change in TFP from 2011 to 2018. Moreover, the evolution of TFP is highly similar to that of TP, which implies the TFP growth is driven mainly by TP in evolution, which differ from the present in Table 4. The TFP growth is lowest in 2015, when China began to reduce the budget growth rate in health sector. TEC changes little during the sample period, which implies that the reforms on public hospitals have little effect on promoting the efficiency of public hospitals. SEC increases first and then decreases to be negative, which implies that the scale of public hospitals is apart from optimal scale.

Thanks to the application of aggregate DDF, we can further identify each region’s contribution to the overall TFP growth of the public hospital system in China. The results in Table 6 suggest that the highest TFP growth rate is observed for Hubei, whereas the lowest one is observed for Hebei. It should be noted that the negative TFP growth regions include Hebei, Fujian, Jiangxi, Henan and Gansu, which are relatively underdeveloped. There are two regions with negative TEC, namely Fujian and Qinghai. These regions require revision of the public hospital system as they departed from the best practice frontier over the time. The negative TP was observed for 16 regions. These regions saw a declining production frontier which indicates abandonment of the best practices. More attention to technological innovation and staff training are needed in the frontier regions.

**Table 6:** The average annual TFP growth rates and its components (%, 2011–2018).

Province	TFP	TEC	TP	SEC
**Eastern**	0.44	0.16	0.26	0.02
Beijing	0.09	0.05	-0.01	0.05
Tianjin	0.00	0.00	-0.01	0.01
Hebei	-0.25	0.00	-0.50	0.24
Liaoning	0.21	0.09	0.09	0.04
Shanghai	0.14	0.00	0.12	0.02
Jiangsu	0.19	0.05	0.14	-0.01
Zhejiang	0.07	0.00	-0.03	0.10
Fujian	-0.02	-0.03	-0.03	0.04
Shandong	-0.13	0.00	0.20	-0.33
Guangdong	0.14	0.00	0.30	-0.16
Hainan	0.01	0.00	-0.01	0.01
**Inland**	0.59	0.32	0.37	-0.10
Shanxi	0.18	0.10	0.02	0.06
Jilin	0.07	0.08	-0.05	0.04
Heilongjiang	0.06	0.06	-0.05	0.05
Anhui	0.12	0.02	0.08	0.02
Jiangxi	-0.03	0.00	-0.02	0.00
Henan	-0.13	0.00	0.07	-0.20
Hubei	0.22	0.05	0.20	-0.03
Hunan	0.09	0.00	0.13	-0.04
**Western**	0.35	0.17	-0.03	0.21
Inner Mongolia	0.05	0.04	-0.05	0.06
Guangxi	0.00	0.01	-0.03	0.02
Chongqing	0.04	0.01	0.01	0.01
Sichuan	0.07	0.00	0.14	-0.07
Guizhou	0.00	0.00	-0.03	0.03
Yunnan	0.02	0.00	-0.02	0.04
Tibet	0.01	0.00	0.01	0.00
Shaanxi	0.12	0.05	0.03	0.05
Gansu	-0.01	0.02	-0.04	0.02
Qinghai	0.00	-0.01	0.00	0.01
Ningxia	0.01	0.00	0.00	0.01
Xinjiang	0.05	0.05	-0.03	0.03

In order to deliver meaningful policy guidelines, one needs to identify the major drivers of TFP change. The linear model is established to relate the TFP change to the explanatory factors:

$$p_{it}=\beta_{0}+\beta_{i}\cdot x_{it}+\varepsilon_{it}$$ (10)

where indexes *i* and *t* denote region and time respectively; *p*_*it*_ denotes TFP, TEC, TP or SEC as a dependent variable; *x*_*i*_ denotes determinants of the TFP change; *β* is the vector of the regression coefficients; *ε*_*it*_ is the error term.

The explanatory variables described in “Data” enter the linear model as regressors. The results of the regression analysis are presented in Table 7. *Oprice* measures the outpatient service price and has a positive coefficient on TEC, which indicates increase in the price of outpatient service will result in improvement in technical efficiency. The possible reason is that the relatively low prices of outpatient services currently prevail in China. They result in patients' excessive demand for outpatient services and inefficient use of medical resources. *Iprice* is a measure of inpatient service price. It can be used to verify the concerns that high price of inpatient service leads to the excessive economic burden on the patients and delay their visits to hospitals. Indeed, the negative coefficients on TFP, TEC and SEC corroborate this hypothesis.

**Table 7:** Determinants of regional hospital efficiency in China.

Variable	*TFP*	*TEC*	*TP*	*SEC*
***Intercept***	0.0059335 ***	0.0021533 ***	0.0017248	0.0020554 **
	(0.0013)	(0.0005)	(0.0015)	(0.0008)
***Oprice***	0.0000055	0.0000035 **	-0.0000045	0.0000066 **
	(0.0000)	(0.0000)	(0.0000)	(0.0000)
***Iprice***	-0.0000002 ***	-0.0000001 *	-0.0000001	-0.0000001 **
	(0.0000)	(0.0000)	(0.0000)	(0.0000)
**Insura**	-0.0005200	-0.0001900	0.0001616	-0.0004915
	(0.0005)	(0.0002)	(0.0006)	(0.0003)
***Subsid***	0.0000146	-0.0008201	-0.0016977	0.0025324 **
	(0.0017)	(0.0006)	(0.0019)	(0.0010)
***Compet***	0.0004960	-0.0004566	0.0002268	0.0007257
	(0.0011)	(0.0004)	(0.0013)	(0.0007)
***Qualiaβ6***	0.0098687 **	-0.0032518 *	0.0141122 **	-0.0009917
	(0.0049)	(0.0018)	(0.0056)	(0.0030)
***Demand***	-0.0205916 ***	-0.0051469 ***	-0.0028772	-0.0125675 ***
	(0.0047)	(0.0017)	(0.0054)	(0.0029)
***R* square**	0.384	0.341	0.058	0.340
	(0.00165)	(0.00061)	(0.00189)	(0.0010)

Note: Figures in parentheses are standard deviations.

***, **, and * indicate significance at the 1%, 5%, and 10% statistical levels, respectively.

*Insura* has no significant effect on TFP and its components, which indicates that it is not likely to improve the TFP of public hospitals by increasing the coverage of medical insurance. Public hospitals have an information advantage over insurance institutions and patients. Furthermore, Chinese government stresses the financial burden of patients and neglects their health responsibility for medical services by continuously reducing the deductible and copayment ratio. These eventually lead to the inefficient use of medical resources.

*Subsid* has no significant effect on TFP and its component, except for SEC, which is the reason that government is suggested to change subsidies by institutions into subsidies by workload. However, the economies of scale need to be assessed in this context. *Compet* has no significant effect on TFP and its components. In the current state of China's medical service market, it may not be reasonable to improve the efficiency of public hospitals by competing with private hospitals due to public hospitals’ advantage over the private hospitals in service quality, scope and market share. *Qualia* is the only variable that has significant impact on TP, because a large part of the quality of medical services is reflected in the technology of medical equipment and staff. The estimated coefficient on *Demand* is negative in all cases suggesting that a province with a higher ratio of population aged 0–14 and above 65 years implies greater demand on hospital services, resulting in lower TFP growth rates. The reduced deductibles lead to excessive demand due to insurance reimbursement plan, the low price of outpatient service and the high price of inpatient service.

## Discussion

Chinese public hospitals experience TFP growth of 1.38% per annum during 2011–2018. The results are similar to 1.21% reported by Shen and Valdmanis [22] and 1.87% provided by Chen et al. [21]. Therefore, the regional public hospitals system is being improved gradually along with development of the whole healthcare system.

We found that the price of inpatient service has negative effect on TFP, TEC and SEC, which indicates that reducing the price will improve the efficiency of the public hospitals. Hu et al. [14] reported similar results and argued that provinces with high prices may reduce the demand for medical services. However, we also found that increasing the price of outpatient services can improve the efficiency of regional public hospitals, which is different from Hu et al. [14]. The possible explanation is that Hu et al. took the average weighted expenditure of outpatients and inpatients as the price and ignored the possible substitution relationship between outpatient and inpatient service due to different price levels. Insurance has no significant effect on TFP and its components, which is different from Hu et al. [14]. They found the coverage rate of insurance may improve efficiency of health sector significantly. A possible reason for this difference is that the periods covered vary across these studies. Specifically, our paper focuses on the last medical reform (2011–2018), whereas Hu et al.’s study focuses on the marketization stage (1998–2007).

The public subsidies have no significant effect on the TFP (with exception for SEC), which is different from Cheng et al. [13]. Indeed, they found a negative impact of subsidies on efficiency. This was explained by assuming that hospitals with higher subsidy rates experienced low efficiency due to moral hazard problem.

Development of China’s healthcare system remains an important dimension of the societal change as it is the case in many countries [34]. Especially, the rural population requires additional attention [35]. The sustainability of the healthcare system also needs to be considered in the long run [36].

There are limitations to the current study that should be acknowledged. First, the regionals are taken as the evaluation unit. Accordingly, the proposed policies and suggestions are relative to the macro-level and cannot be directly imposed to improve the efficiency of individual public hospitals. Second, due to the time lag of policy effect, the policy effect may be over- or underestimated in the relatively short period from 2011 to 2018. Third, although the method used in this paper has some advantages, it has some well-known problems of the nonparametric methods, such as lack of statistical inference. In addition, we just examined the efficiency changes of regional public hospitals after the implementation of the novel health care reform, but did not compare to that efficiency and productivity of public hospitals before the 2009 reforms. Therefore, a comparison of the periods before and after the implementation of the policy reform can be provided in further research.

## Conclusion

In this paper, we proposed a novel framework for the additive decomposition of the TFP based on the aggregate DDF, and used it to analyze TFP change in Chinese public hospitals over 2011–2018. This method allows us to identify the contribution of each region to the growth in the TFP and its components. We find the average annual TFP growth rate of 1.38%, which is mainly driven by technical efficiency.

The determinants of the TFP change were identified by adopting the regression approach. We found that the prices of outpatient and inpatient services have different impact on the efficiency of regional public hospitals: price of the outpatient services should be increased and the price of the inpatient services should be reduced in order to improve efficiency. The public subsidy and insurance penetration had no significant impact on the TFP change. The increase in competitive environment (i.e., increasing importance of the private hospitals) showed no effect of the TFP change.

## Supporting information

S1 Data (XLSX)
